# The general anaesthetic etomidate inhibits the excitability of mouse thalamocortical relay neurons by modulating multiple modes of GABA_A_ receptor-mediated inhibition

**DOI:** 10.1111/ejn.12601

**Published:** 2014-04-29

**Authors:** Murray B Herd, Jeremy J Lambert, Delia Belelli

**Affiliations:** Division of Neuroscience, Medical Research Institute, University of Dundee, Ninewells Hospital and Medical SchoolDundee, DD1 9SY, UK

**Keywords:** nucleus reticularis, phasic inhibition, spill-over inhibition, thalamus, tonic inhibition

## Abstract

Modulation of thalamocortical (TC) relay neuron function has been implicated in the sedative and hypnotic effects of general anaesthetics. Inhibition of TC neurons is mediated predominantly by a combination of phasic and tonic inhibition, together with a recently described ‘spillover’ mode of inhibition, generated by the dynamic recruitment of extrasynaptic γ-aminobutyric acid (GABA)_A_ receptors (GABA_A_Rs). Previous studies demonstrated that the intravenous anaesthetic etomidate enhances tonic and phasic inhibition in TC relay neurons, but it is not known how etomidate may influence spillover inhibition. Moreover, it is unclear how etomidate influences the excitability of TC neurons. Thus, to investigate the relative contribution of synaptic (α1β2γ2) and extrasynaptic (α4β2δ) GABA_A_Rs to the thalamic effects of etomidate, we performed whole-cell recordings from mouse TC neurons lacking synaptic (α1^0/0^) or extrasynaptic (δ^0/0^) GABA_A_Rs. Etomidate (3 μm) significantly inhibited action-potential discharge in a manner that was dependent on facilitation of both synaptic and extrasynaptic GABA_A_Rs, although enhanced tonic inhibition was dominant in this respect. Additionally, phasic inhibition evoked by stimulation of the nucleus reticularis exhibited a spillover component mediated by δ-GABA_A_Rs, which was significantly prolonged in the presence of etomidate. Thus, etomidate greatly enhanced the transient suppression of TC spike trains by evoked inhibitory postsynaptic potentials. Collectively, these results suggest that the deactivation of thalamus observed during etomidate-induced anaesthesia involves potentiation of tonic and phasic inhibition, and implicate amplification of spillover inhibition as a novel mechanism to regulate the gating of sensory information through the thalamus during anaesthetic states.

## Introduction

The ability of general anaesthetics to impair consciousness, and induce analgesia, amnesia and immobility, has been exploited clinically for over a century (Rudolph & Antkowiak, 2004). Yet, despite their routine clinical use, the neuroanatomical substrates of anaesthetic actions remain enigmatic, with several components of the circuitry regulating the sleep–wake cycle implicated to date (Franks, 2008). Nevertheless, electrophysiological, neuroimaging and modelling studies consistently pinpoint the thalamus as an important neural locus for anaesthetic-induced hypnosis (Angel, 1991; Fiset *et al*., 1999; Alkire *et al*., 2000; White & Alkire, 2003; Ching *et al*., 2010; Andrada *et al*., 2012). Such observations are consistent with the classical role of the thalamus in controlling transitions between conscious states (Steriade *et al*., 1993).

At the cellular level, γ-aminobutyric acid (GABA)_A_ receptors (GABA_A_Rs) represent key molecular targets for several general anaesthetics, including the intravenous agents etomidate and propofol (Hales & Lambert, 1991; Orser *et al*., 1994; Uchida *et al*., 1995). Importantly, anaesthetic-induced modulation of inhibition may be influenced by the molecular identity of GABA_A_Rs (Belelli *et al*., 1996; Bonin & Orser, 2008), which are composed of five subunits drawn from a pool of 19 isoforms (Olsen & Sieghart, 2009). Etomidate is unique in this respect, displaying selectivity for β2- or β3-containing receptors vs. those incorporating the β1 subunit (Hill-Venning *et al*., 1997). Subunit composition additionally influences the functional properties and spatiotemporal expression of GABA_A_Rs. Thus, synaptic GABA_A_Rs, which mediate brief phasic inhibition, are composed of α*x*β*x*γ2 subunits, whereas receptors incorporating a δ rather than a γ2 subunit, are localized to extra- or peri-synaptic locations, and mediate persistent tonic inhibition (Farrant & Nusser, 2005). However, a substantial number of GABA_A_Rs with a typically ‘synaptic’ subunit composition also exist extrasynaptically, although the functional role of such receptors remains to be established (Kasugai *et al*., 2010). δ-GABA_A_Rs have been proposed as important general anaesthetic targets, a suggestion supported by their abundant expression in brain regions implicated in some anaesthetic behaviours, including thalamocortical (TC) relay nuclei (Belelli *et al*., 2005; Jia *et al*., 2005). However, while etomidate enhances both phasic and tonic currents recorded from TC neurons (Belelli *et al*., 2005), it is unknown how these respective actions influence the excitability of relay neurons.

Recently, we demonstrated that in addition to generating tonic inhibition, δ-GABA_A_Rs of TC neurons may be recruited via a spillover-like mechanism to prolong phasic inhibition generated in response to presynaptic spike bursts (Herd *et al*., 2013). This observation may have particular relevance to anaesthetic effects in thalamus, given that nucleus reticularis thalami (nRT) neurons, which provide the major source of GABA-ergic innervation to TC neurons, exhibit prominent spike bursts during unconscious states. Whether and how anaesthetics may influence this ‘burst-mediated spillover’ mode is not known.

Here, we investigated the relative contribution of synaptic and extrasynaptic GABA_A_Rs to the inhibitory effects of etomidate on relay neuron output. Using transgenic mice in which TC relay neurons lack synaptic (α1^0/0^, Peden *et al*., 2008) or extrasynaptic (δ^0/0^, Herd *et al*., 2009) GABA_A_Rs, we report that, although enhancement of tonic inhibition dominates the inhibitory effect of etomidate on TC neuron excitability, the synaptic α1βγ2 GABA_A_R population also significantly influences the effects of the anaesthetic. Moreover, we demonstrate that etomidate greatly prolongs burst-mediated spillover inhibition, providing a novel mechanism whereby a putatively relevant network may be influenced by anaesthetics.

## Materials and methods

### Use of α1^0/0^ and δ^0/0^ mice as models to investigate the thalamic effects of etomidate

Compensatory adaptations represent a significant caveat to the interpretation of experiments utilizing constitutive knockout mice. Indeed, biochemical and immunohistochemical evidence revealed compensatory up-regulation of α2 and/or α3 subunits in the neocortex and cerebellum of α1^0/0^ mice (Sur *et al*., 2001; Kralic *et al*., 2002a,b2002b). However, in the thalamus of α1^0/0^ mice, the ablated α1 subunits are not replaced by α2 or α3 subunits (Kralic *et al*., 2006) and α3 expression remains unaltered in the presynaptic nRT (Kralic *et al*., 2006). Thus, at the functional level, the lack of any clear upregulation of the remaining palette of GABA_A_R subunits is reflected by a complete lack of miniature inhibitory postsynaptic currents (mIPSCs) in mature ventrobasal (VB) neurons, but unaltered mIPSC properties in the nRT (Peden *et al*., 2008). Moreover, extrasynaptic GABA_A_Rs do not appear to compensate for the lost phasic inhibition, as tonic inhibition is not significantly altered in α1^0/0^ VB neurons, at least at the limited age range studied here (Herd *et al*., 2009). In the δ^0/0^ mouse (Mihalek *et al*., 1999), increased expression of γ2 subunits has been reported (Tretter *et al*., 2001; Peng *et al*., 2002). Importantly, however, receptors incorporating α1, α2, α3 or α5 subunits do not appear to substitute for the lost δ-containing receptors (Peng *et al*., 2002). In agreement, we did not observe any clear alterations in the properties of relay neuron mIPSCs that could compensate for the greatly reduced tonic inhibition in δ^0/0^ mice (Herd *et al*., 2009). Thus, the lack of clear adaptive changes in the properties of phasic and tonic inhibition at the thalamic level in the α1^0/0^ and δ^0/0^ mice validates their utility as models to investigate the influence of etomidate on relay neuron excitability.

The α1^0/0^ and δ^0/0^ mice were generated on a mixed C57BL6/J-129SvEv (α1^0/0^) or single C57BL6 (δ^0/0^) background at the Merck Research Laboratories at the Neuroscience Research Centre in Harlow and at the University of Pittsburgh, respectively, as described previously (Mihalek *et al*., 1999; Sur *et al*., 2001). Brain slices were prepared from the first two generations of α1^0/0^ and δ^0/0^ and their respective wild-type (WT) mice from breeding pairs derived from the corresponding heterozygous mice bred at the University of Dundee. Measures of inhibition (phasic and tonic) and neuronal excitability [input resistance (IR) and action potential rheobase] did not differ between α1^+/+^ and δ^+/+^ (data not shown). Results obtained from the respective WT strains were thus pooled.

### Slice preparation

Animals were killed by cervical dislocation in accordance with Schedule 1 of the UK Government Animals (Scientific Procedures) Act 1986. Thalamic slices were prepared from mice of either sex (postnatal days 16–24) according to standard protocols (Herd *et al*., 2009). Dissected brains were sliced horizontally (300–350 μm) in an ice-cold sucrose-based cutting solution, using a Leica VT1000S vibratome (Nussloch, Germany). The slices were incubated at room temperature (20–23 °C) for a minimum of 1 h prior to recording in an oxygenated, extracellular solution (ECS) containing (in mm): 126 NaCl, 2.95 KCl, 26 NaHCO_3_, 1.25 NaH_2_PO_4_, 2 CaCl_2_, 10 d-glucose and 2 MgCl_2_ (pH 7.4; 300–310 mOsm).

### Electrophysiology

Electrophysiological recordings were performed from thalamic slices maintained in warmed ECS (30 °C), using an Axopatch 200B amplifier (Molecular Devices, Sunnyvale, CA, USA). Thalamocortical relay neurons of the VB complex were visually identified with an Olympus BX51 microscope (Olympus, Southall, UK), equipped with differential interference contrast/infrared optics and a charge-coupled device camera. Whole-cell voltage- and current-clamp recordings from VB neurons were obtained using patch pipettes prepared from thick-walled borosilicate glass (King Precision Glass, Claremont, CA, USA) using a Narishige PC-10 vertical puller, and had open tip resistances of 4–6 MΩ when filled with a solution containing [in mm): 130 K-gluconate, 2 KCl, 2 NaCl, 10 HEPES (4-(2-hydroxyethyl)-1-piperazineethanesulfonic acid), 0.2 EGTA (ethylenediaminetetraacetic acid), 2 Mg adenosine triphosphate, 0.5 Na guanosine-5′-triphosphate, 10 Tris-phosphocreatine, adjusted to pH 7.2–7.3 with KOH, 280–290 mOsm. Series resistance was intermittently monitored, compensated for (up to 80% for voltage-clamp, 100% for current-clamp) and recordings discarded if values changed by more than 25%, or increased beyond 25 MΩ. Voltage-clamp recordings were performed at a holding potential of −50 mV (GABA *E*_rev_ ∼ −85 mV), and thus responses mediated by GABA_A_Rs were outwardly directed. Inhibitory responses were evoked in VB neurons using a bipolar tungsten electrode (World Precision Instruments, Stevenage, UK), placed within the borders of the nRT. Stimuli were delivered (20-μs duration, 0.1 Hz) using a Digitimer DS3 Isolated Stimulator (Digitimer, Welwyn Garden City, UK), with stimulus intensity adjusted to the minimum value that avoided failures, but still generated IPSC bursts of relatively consistent amplitude [i.e. for each recording, the excitatory IPSC (eIPSC) peak amplitude standard deviation (SD) was <15% of the mean peak amplitude obtained from that cell; WT = 8.5 ± 1.4%, *n* = 7; α1^0/0^ = 10.5 ± 1.4%, *n* = 7; δ^0/0^ = 8.9 ± 0.8%]. At the stimulation intensities utilized, the number of peaks observed in response to each stimulus was relatively consistent from trial to trial (SD = 0.94 and 1.30 for WT and δ^0/0^, respectively). Although we find GABA_B_R activation is minimal under these recording conditions/stimulation parameters (data not shown), we performed the experiments in the presence of CGP 55845 (1 μm) to block GABA_B_Rs. Holding currents (*I*_hold_) were continuously monitored throughout voltage-clamp recordings to determine the effect of etomidate on tonic inhibition. Current-clamp recordings were also performed to determine the effect of burst-mediated inhibition on VB neuron spiking. Thus, evoked inhibitory postsynaptic potentials (eIPSPs) were evoked at regular intervals (0.09–0.11 Hz) during tonic trains of action potentials. Tonic spike trains were generated by depolarizing the recorded neuron to a supra-threshold membrane potential (*V*_m_) using depolarizing direct current (DC) injection delivered via the recording electrode. The amplitude of injected DC was carefully adjusted to maintain a tonic spike frequency of ∼4–8 Hz. In this paradigm, eIPSPs generated a transient cessation of action potential discharge. eIPSPs were also recorded at or near the resting potential, and in some cells a rebound low-threshold Ca^2+^ potential was observed, which was commonly crowned by a high-frequency spike burst. In such recordings, the latency to rebound, relative to eIPSP onset, was determined for each event before and after etomidate application. VB neuron excitability was also examined in current-clamp experiments by monitoring action potential output in response to a family of current steps. The stimulation protocol consisted of a set of hyperpolarizing and depolarizing current pulses (−200 to +300 pA, 400-ms duration, in increments of 50 pA, every 15 s), delivered via the recording electrode before and after the bath application of etomidate. Current stimulations were delivered from a holding potential of ∼−66 mV to isolate the tonic spike mode during depolarizing stimulations. The number of action potentials generated in response to positive current steps, and within low-threshold Ca^2+^ current (*I*_T_)-mediated rebound bursts following release from hyperpolarization, were counted for each condition. Membrane IR was calculated according to Ohms's law following delivery of small (−20 pA, 300 ms) hyperpolarizing current steps from the resting membrane potential. A measured liquid junction potential of 11 mV was corrected offline. Recordings were filtered at 2 or 5 kHz for voltage- and current-clamp recordings, respectively, and acquired directly to PC using a NI-DAQmx analog-to-digital interface (National Instruments UK, Newbury, UK) for subsequent offline analysis.

### Data analysis

All electrophysiological data were analysed offline using the Strathclyde Electrophysiology Software, WinEDR/WinWCP (J. Dempster, University of Strathclyde, UK) software package. For voltage-clamp experiments, eIPSCs were analysed with respect to peak amplitude, 10–90% rise times and charge transfer. The decay time course following the final peak of multi-peak eIPSCs was adequately described by fitting a double exponential function (*y*(*t*) = *A*_1_*e*^(−*t*/τ1)^ + *A*_2_*e*^(−*t*/τ2)^) using the least squares method, where *A* is amplitude, *t* is time and τ is the decay time constant. Analysis of the SD of residuals and use of the *F*-test was used to compare goodness of fit, confirming an improved fit of the decay was achieved when employing a double- rather than a mono-exponential function. Thus, a weighted decay time constant (τ_w_) was also calculated according to the equation, τ_w_ = τ_1_*P*_1_ + τ_2_*P*_2_, where τ_1_ and τ_2_ are the decay time constants of the first and second exponential functions and *P*_1_ and *P*_2_ are the proportions of the synaptic current decay described by each component. To determine the effect of etomidate on VB tonic inhibition, etomidate-induced outward shifts of the holding current (*I*_hold_) were quantified by sampling sequential 51.2-ms epochs for a 2-min period before, and after application of etomidate (Belelli *et al*., 2005). Epochs containing evoked or spontaneous synaptic currents were excluded. The mean holding current in each condition was determined from Gaussian fits to the distribution of holding current values sampled from each epoch.

For current-clamp experiments investigating the effect of etomidate on VB neuron excitability, input–output curves were constructed by plotting the number of action potentials generated as a function of stimulus amplitude. To quantify etomidate-induced shifts in input–output curves, data were fit with a Boltzman sigmoidal curve according to the equation *y*(*x*) = *N*_max_/1 + e^(*X* – *X*^_50_^)/*S*^ + *N*_max_), where *N*_max_ is the number of action potentials generated at the maximum stimulus amplitude (in this case 300 pA), *X* is the stimulus amplitude, *X*_50_ is the stimulus amplitude producing half-maximal spike output (hereafter ‘EA_50_’) and *S* is the slope factor. For experiments investigating the effect of eIPSPs on tonic spike trains, inter-spike intervals (ISIs) were measured throughout the spike train. For baseline measurements, ISIs were determined for a 5-s period preceding each eIPSP (minimum of three stimulations) to ensure spike rates recovered to stable frequencies following inhibition. The ISI during an IPSP was simply the interval between the final spike prior to delivery of an eIPSP and the first spike following recovery from inhibition. The transient suppression of tonic spike activity induced by an eIPSP was observed as an immediate and obvious increase in the ISI (see Figs2 and 3).

All results are reported as the arithmetic mean ± standard error of the mean (SEM). Statistical significance of the mean data was assessed with a Student's *t* test (paired, or unpaired), one-way analysis of variance (anova) or a two-way mixed-design anova as appropriate, using the SPSS (SPSS Inc., Chicago, IL USA) software package. Sphericity was assessed with Mauchly's test, and degrees of freedom were adjusted using a Greenhouse–Geisser correction if sphericity was violated. Post-hoc multiple comparisons were conducted using Tukey's test. Unless explicitly stated, statistical significance was assessed using Student's *t*-test. Boltzmann curve fits were performed in Origin v7 (OriginLab, Northampton, MA, USA).

### Salts and drugs

*R*-(+)-etomidate (supplied by Merck, Glasgow, UK) was prepared as a concentrated aqueous stock and diluted 1000-fold in ECS to a final concentration of 3 μm. CGP 55835 (Abcam, Cambridge, UK) was prepared in dimethyl sulphoxide (DMSO) as a 1000-fold concentrated stock solution and diluted in ECS to the final desired concentration. The final DMSO concentration (0.1%) had no effect on any of the measured parameters (data not shown). Etomidate was applied via the perfusion system (2–4 mL/min) and allowed to infiltrate the slice for a minimum of 10 min. All other salts and drugs were obtained either from Sigma-Aldrich (Poole, UK), Tocris Bioscience (Bristol, UK) or VWR (Lutterworth, UK).

## Results

### Etomidate prolongs eIPSCs by potentiating synaptic and extrasynaptic GABA_A_Rs

In VB neurons, etomidate prolongs mIPSCs and enhances tonic currents mediated by α1β2γ2- and by α4β2δ GABA_A_Rs, respectively (Belelli *et al*., 2005). However, our recent demonstration that thalamic δ-GABA_A_Rs may influence the kinetics of action potential-dependent phasic inhibition, particularly in response to spike bursts (Herd *et al*., 2013), provides an additional route whereby etomidate may influence VB inhibition. Thus, we investigated the effects of etomidate on electrically evoked IPSCs generated in response to extracellular stimulation of the nRT of WT, α1^0/0^ and δ^0/0^ mouse brain slices.

**Figure 1:**
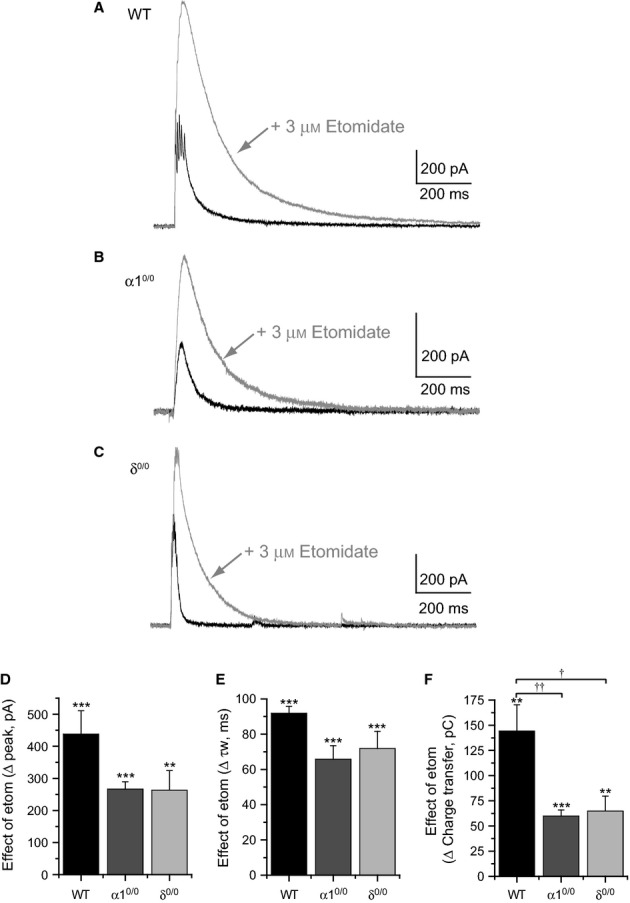
Etomidate prolongs the duration of evoked phasic inhibition in VB neurons by potentiating synaptic and extrasynaptic GABA_A_ receptors. (A–C) Superimposed, representative, evoked IPSCs recorded from WT (A), α1^0/0^ (B) and δ^0/0^ (C) VB neurons under control conditions (black) and in the presence of 3 μm etomidate (grey). Currents were evoked by extracellular stimulation of the nRT (single shocks, 20 μs duration). (D–E) Bar graphs illustrating the effect of etomidate on eIPSC peak amplitude (D), τ_w_ (E) and charge transfer (F) in WT (black bars, *n* = 7), α1^0/0^ (dark grey bars, *n* = 7) and δ^0/0^ (light grey bars, *n* = 8) thalamic slices. **P <* *0.01, ***P <* *0.001 vs. control, paired *t*-test. ^†^*P *<* *0.05, ^†^^†^*P *<* *0.01 vs. WT, mixed anova, Tukey's post-hoc test. Etom, etomidate.

In voltage-clamp recordings utilising K^+^-gluconate-based pipette solutions (GABA_A_R *E*_rev_ ∼ −85 mV, *V*_h_ = −50 mV), extracellular stimulation of the nRT in WT brain slices generated outwardly directed eIPSCs composed of multiple peaks, and a bi-exponential decay phase following the final peak (Table1, Fig.1A). The multi-peak nature of the eIPSCs is consistent with the generation of *I*_T_-dependent spike bursts in presynaptic neurons. Application of etomidate (3 μm) greatly increased the eIPSC peak amplitude (Table2, Fig.1A and D, *n *= 7, *P *<* *0.001), and τ_w_ (Table2, Fig.1A and E, *P *< 0.001), resulting in a significant enhancement of eIPSC charge transfer (Table2, Fig.1A and F, *P = *0.001). Consistent with our previous report (Herd *et al*., 2013), robust residual eIPSCs were recorded from α1^0/0^ VB neurons, despite the absence of synaptic GABA_A_Rs (Table1, Fig.1B). The slow kinetics of the rising and decaying phase of α1^0/0^ eIPSCs, together with their sensitivity to modulation by the δ-selective compound DS2 (Wafford *et al*., 2009; Herd *et al*., 2013; Jensen *et al*., 2013), indicate that such residual events are mediated by recruitment of δ-containing extrasynaptic (e)GABA_A_Rs in response to nRT spike bursts. Importantly, acute application of etomidate greatly enhanced the peak amplitude (Table2, Fig.1B and D, *n *= 7, *P *<* *0.001) and τ_w_ (Table2, Fig.1B and E, *P *< 0.001) of α1^0/0^ eIPSCs, leading to a significant increase in their charge transfer (Table2, Fig.1F, *P *< 0.001). However, the overall effect of etomidate on eIPSC charge transfer was reduced for α1^0/0^ relative to WT neurons (Fig.1F, drug × genotype interaction, *F*_2,19_ = 7.21*, P = *0.005, mixed anova), suggesting that a significant proportion of the effects of etomidate on WT eIPSCs are mediated by synaptic GABA_A_Rs. In support, etomidate also enhanced eIPSCs recorded from δ^0/0^ neurons (Fig.1C). In agreement with previous findings (Herd *et al*., 2013), eIPSCs recorded from δ^0/0^ VB neurons exhibited faster decay kinetics, due to the abolished eGABA_A_R component normally engaged by neurally released GABA acting on WT neurons (Table1). Nevertheless, despite the absence of the eGABA_A_R contribution to eIPSC properties, etomidate (3 μm) significantly increased eIPSC peak amplitude (Table2, Fig.1C and D, *n *= 8, *P *= 0.003), τ_w_ (Table2, Fig.1C and E, *P *< 0.001) and charge transfer (Table2, Fig.1F, *P = *0.003). However, as observed for α1^0/0^ recordings, deletion of the δ subunit significantly reduced the overall effect of etomidate on eIPSC charge transfer relative to WT (Fig.1F, drug × genotype interaction, *F*_2,19_ = 7.21, *P = *0.005, mixed anova).

**Table 1:** Comparison of eIPSC properties recorded from WT, α1^0/0^ and δ^0/0^ mice

	WT (*n *=* *16)	α1^0/0^ (*n *=* *14)	δ^0/0^ (*n *=* *10)
No. of peaks/eIPSC	7.6 ± 0.4	N/D	7.2 ± 0.4
Mean frequency of peaks/eIPSC (Hz)	269.8 ± 16.2	N/D	270.9 ± 14.0
Peak amplitude (pA)	604.1 ± 70.7	142.9 ± 22.7***	493.2 ± 58.6
Rise Time to 1st peak (ms)	1.1 ± 0.2	14.6 ± 0.9***	1.1 ± 0.1
Charge transfer (pC)	39.0 ± 5.5	11.5 ± 2.1***	16.1 ± 2.3**
T_50_ (ms)	28.2 ± 1.7	46.2 ± 6.0**	17.6 ± 1.0***
τ_1_ (ms)	28.2 ± 2.5	44.9 ± 5.8**	9.9 ± 0.7**
τ_2_ (ms)	219.0 ± 18.8	233.9 ± 19.8	50.8 ± 3.5***
% τ_1_	82.0 ± 3.6	84.8 ± 2.9	72.5 ± 2.3
τ_w_ (ms)	58.3 ± 6.3	76.3 ± 11.3	21.5 ± 2.1*

* *P*<0.05

** *P*<0.01

*** *P*<0.001

vs. WT, one-way anova, Tukey's post-hoc test. N/D, not determined.

**Table 2:** Comparison of the effect of etomidate on eIPSCs recorded from WT, α1^0/0^ and δ^0/0^ mice

	WT (*n *=* *7)		α1^0/0^ (*n *=* *7)		δ^0/0^ (*n *=* *8)	
	Control	3 μm etomidate	Control	3 μm etomidate	Control	3 μm etomidate
Peak amplitude (pA)	599 ± 101	1037 ± 152***	198 ± 31	465 ± 40***	477 ± 73	740 ± 121**
τ_w_ (ms)	53 ± 7	144 ± 9***	73 ± 14	139 ± 20***	21 ± 3	93 ± 9***
Charge transfer (pC)	40 ± 8	184 ± 32**	17 ± 3	76 ± 8***	16 ± 3	81 ± 17**

** *P*< 0.01

*** *P*< 0.001

paired *t*‐test.

### Etomidate prolongs phasic suppression of VB tonic firing and delays post-inhibitory rebound burst firing

During conscious brain states, thalamocortical relay neurons exist in a relatively depolarized state, and respond to incoming excitatory inputs in the tonic ‘relay’ mode (Steriade *et al*., 1993). Given the shared roles of synaptic and extrasynaptic GABA_A_Rs in regulating the kinetics of inhibitory synaptic transmission (see above), we hypothesized that these receptor populations should combine to regulate the effect of etomidate on phasic inhibition of tonic VB spike output.

**Figure 2:**
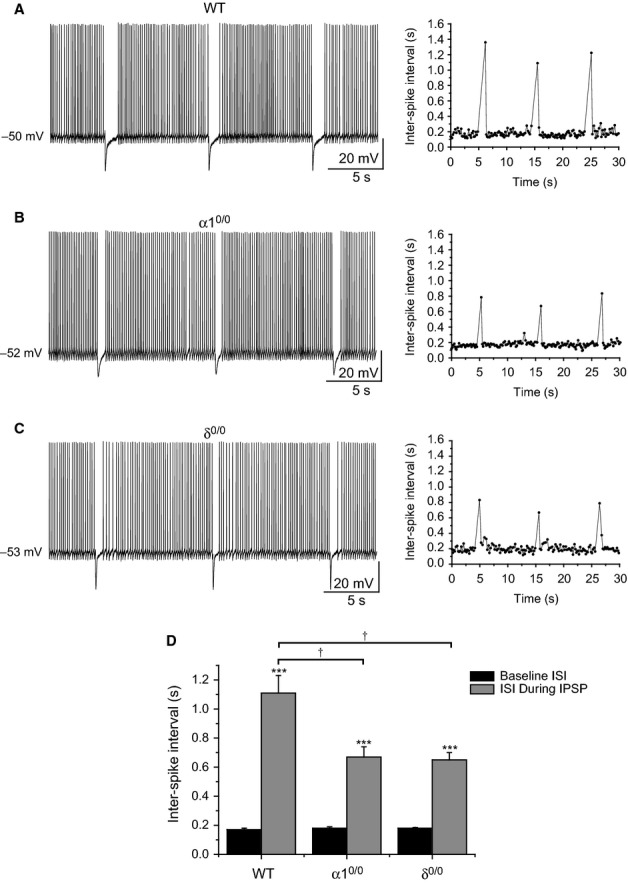
Synaptic and extrasynaptic GABA_A_Rs influence phasic suppression of VB tonic firing. (A–C) Representative current-clamp recordings illustrating the suppression of tonic action potential discharge induced by evoked IPSPs in WT (A), α1^0/0^ (B) and δ^0/0^ (C) thalamic slices. The plots to the right of each trace depict the inter-spike interval (ISI) over the course of the illustrated recordings, and demonstrate the increase in ISI (i.e. spike suppression) during delivery of the eIPSP. Evoked IPSPs were generated by extracellular stimulation of the nRT. (D) Bar graph comparing the effect of eIPSPs on ISI during tonic spike trains recorded from WT (*n* = 13), α1^0/0^ (*n* = 12) and δ^0/0^ (*n* = 8) VB neurons. ***P <* *0.001 vs. baseline ISI, paired *t*-test. ^†^*P *<* *0.05 vs. WT, mixed anova, Tukey's *post hoc* test.

**Figure 3:**
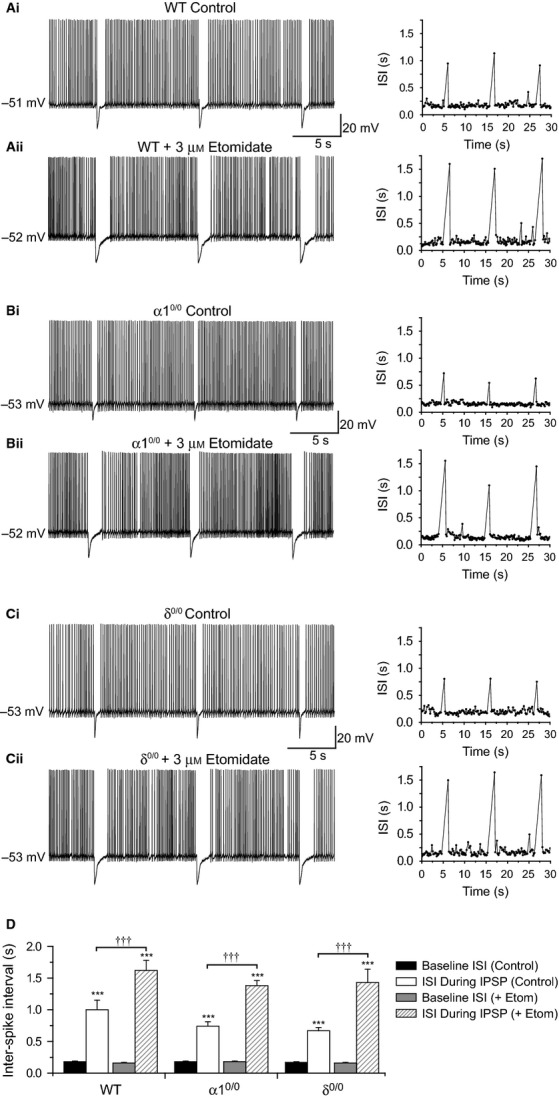
Etomidate prolongs phasic suppression of VB tonic firing by potentiating synaptic and extrasynaptic GABA_A_Rs. (A–C) Whole-cell current-clamp recordings illustrating the suppression of VB tonic firing under control conditions (Ai–Ci), and in the presence of 3 μm etomidate (Aii–Cii), in WT (A), α1^0/0^ (B) and δ^0/0^ (C) thalamic slices. The plots to the right of each trace depict the inter-spike interval (ISI) during the illustrated tonic spike train, and demonstrate blockade of action potentials during the eIPSP. Note that the ISI during the eIPSP is increased in the presence of etomidate across all mouse strains. (D) Bar graph comparing the effect of eIPSPs on the ISI of tonic spike trains, before and after etomidate, in WT (*n* = 8), α1^0/0^ (*n* = 9) and δ^0/0^ (*n* = 6) VB neurons. The bar representations are depicted in the symbol key. ***P <* *0.001 vs. baseline ISI, paired *t*-test. ^†^^†^^†^*P *<* *0.001, ISI during eIPSP after etomidate application vs. control. The increased duration of the eIPSP-induced spike suppression observed in the presence of etomidate is not significantly influenced by mouse genotype (*P* = 0.58, mixed anova).

To explore these questions we performed whole-cell current-clamp recordings of tonic firing from WT, α1^0/0^ and δ^0/0^ slices. VB neurons were slowly depolarized from the resting membrane potential (Table3) by DC injection until they discharged trains of tonic spikes, occurring at ∼4–8 Hz (mean spike frequency: WT = 5.8 ± 0.3 Hz, *n *= 13; α1^0/0^ = 5.7 ± 0.3 Hz, *n* = 12; δ^0/0^ = 5.5 ± 0.1 Hz, *n* = 8; *F*_2,32_ = 0.35, *P *= 0.71, one-way anova). We then evoked IPSPs by extracellular stimulation of the nRT (0.09–0.11 Hz stimulation frequency) at regular intervals during the tonic train, and quantified the duration of the eIPSP-induced suppression of tonic firing across strains (i.e. ISI), before and after etomidate (3 μm) (Figs2 and 3). As illustrated in the representative example in Fig.2A, stimulation of the nRT in WT brain slices evoked reproducible IPSPs, which transiently suppressed tonic spiking of VB neurons (baseline ISI = 173 ± 8 ms; ISI during IPSP = 1106 ± 118 ms; *n* = 13, *P *<* *0.001; Fig.2D). Remarkably, despite the absence of a detectable synaptic receptor population in VB, equivalent stimulation of the nRT in α1^0/0^ slices generated eIPSPs that transiently inhibited tonic spiking, albeit to a lesser extent than in WT tissue (baseline ISI = 176 ± 10 ms; ISI during IPSP = 672 ± 67 ms; *n* = 12, *P *<* *0.001; stimulation × genotype interaction, *F*_2,28_ = 7.04*, P *= 0.003, mixed anova; Fig.2B and D). Similarly, in δ^0/0^ slices, eIPSPs induced a reproducible blockade of VB tonic firing, although the duration of spike suppression was significantly (and similar to α1^0/0^) reduced relative to WT recordings (baseline ISI = 180 ± 5 ms; ISI during IPSP = 650 ± 49 ms; *n* = 8, *P *<* *0.001, stimulation × genotype interaction, *F*_2,28_ = 7.04*, P *= 0.003, mixed anova; Fig.2C and D), consistent with voltage-clamp experiments demonstrating faster eIPSC decay time in the absence of δ-GABA_A_Rs.

**Table 3:** Comparison of resting membrane potential (*V*_m_) and input resistance recorded from WT, α1^0/0^ and δ^0/0^ mice

	WT (*n *=* *38)	α1^0/0^ (*n *=* *19)	δ^0/0^ (*n *=* *27)
*V*_m_ (mV)	–72.5 ± 0.5	–72.0 ± 0.9	–73.5 ± 0.5
Input resistance (MΩ)	288 ± 13	274 ± 22	328 ± 20

Consistent with the robust potentiation of eIPSCs observed in voltage-clamp experiments, etomidate (3 μm) significantly prolonged the duration of tonic firing suppression induced by eIPSPs in WT (ISI during IPSP: control = 1000 ± 148 ms; + etomidate = 1624 ± 161 ms; *n* = 8, *P *<* *0.001; Fig.3A and D), α1^0/0^ (ISI during IPSP: control = 737 ± 67 ms; + etomidate = 1383 ± 78 ms; *n* = 9, *P *<* *0.001; Fig.3B and D) and δ^0/0^ brain slices (ISI during IPSP: control = 670 ± 54 ms; + etomidate = 1427 ± 206 ms; *n* = 6, *P *<* *0.001; Fig.3C and D). Surprisingly, however, the effect of etomidate on eIPSP-induced suppression of tonic firing (i.e. the difference in ISI during an IPSP before and after etomidate application) was not significantly different between strains (Fig.3D, F_2,20_ = 0.56*, P *= 0.58, mixed anova), despite the ablation of either major subcellular population of GABA_A_R.

**Figure 4:**
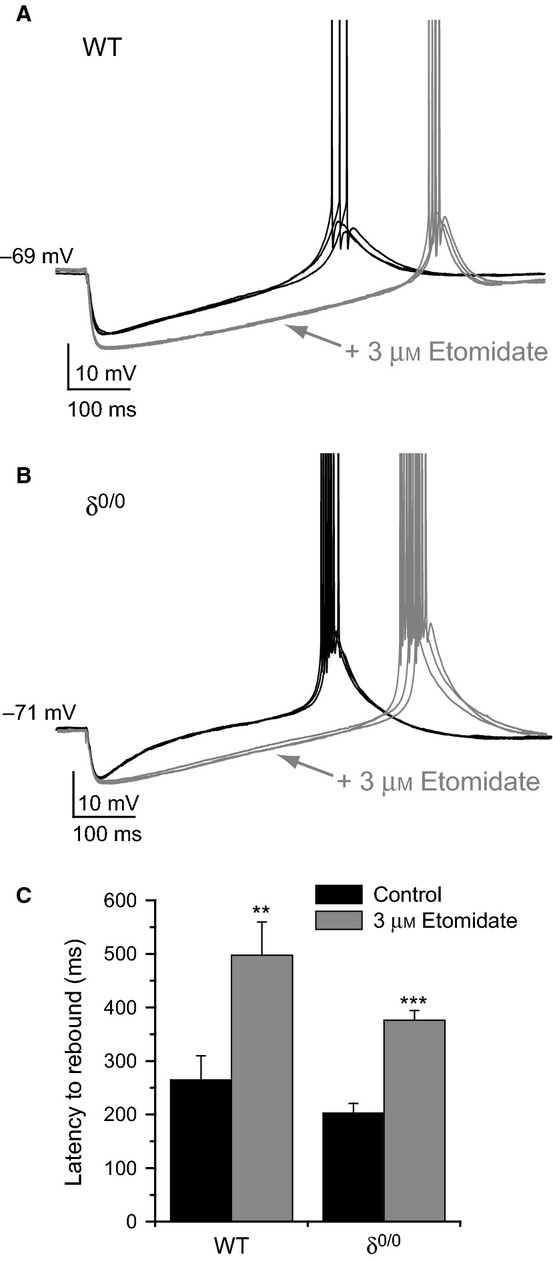
Etomidate delays the timing of post-inhibitory rebound burst firing in VB neurons. (A, B) Exemplar traces from a subset of current-clamp recordings obtained from WT (A) and δ^0/0^ (B) mice in which rebound burst firing was observed at the offset of eIPSPs. The responses to three consecutive stimuli recorded before (black traces) and after (grey traces) application of 3 μm etomidate are superimposed, illustrating the delay to rebound burst firing in the presence of the anaesthetic. Action potentials are truncated for clarity. (C) Summary bar graph comparing the latency to rebound spiking before (black bars) and after etomidate (grey bars) application for WT (*n* = 4) and δ^0/0^ (*n* = 5) VB neurons. **P <* *0.01, ***P <* *0.001, paired *t*-test.

In a subset of these recordings, eIPSPs recorded at or slightly depolarized to the resting membrane potential were of sufficient amplitude to generate a rebound low-threshold Ca^2+^ potential and associated spike burst at the offset of the eIPSP (von Krosigk *et al*., 1993). We therefore investigated the effect of etomidate on the post-inhibitory rebound properties by measuring the latency to rebound. Consistent with the prolongation of evoked phasic inhibition, etomidate significantly delayed the timing of rebound bursts in WT recordings (Fig.4A and C, *n *= 4, *P* = 0.009) at similar *V*_m_ (pre-eIPSP *V*_m_ = −68.3 ± 1.2 and −69.0 ± 1.2 mV for control and etomidate, respectively). Similarly, in δ^0/0^ recordings, the latency to post-inhibitory rebound was significantly increased in the presence of etomidate (Fig.4B and C, *n *= 5, *P *< 0.001) at equivalent *V*_m_ (pre-eIPSP *V*_m_ = −67.8 ± 0.3 and −68.3 ± 0.2 mV for control and etomidate, respectively). eIPSPs recorded from α1^0/0^ slices did not elicit rebound bursts, although in all seven recordings, the duration of eIPSPs recorded from equivalent membrane potentials were prolonged (data not shown). These observations imply that both synaptic and extrasynaptic receptor modulation can influence post-inhibitory rebound timing.

### Deletion of the α1 or δ subunit influences the etomidate-induced enhancement of the VB tonic current

**Figure 5:**
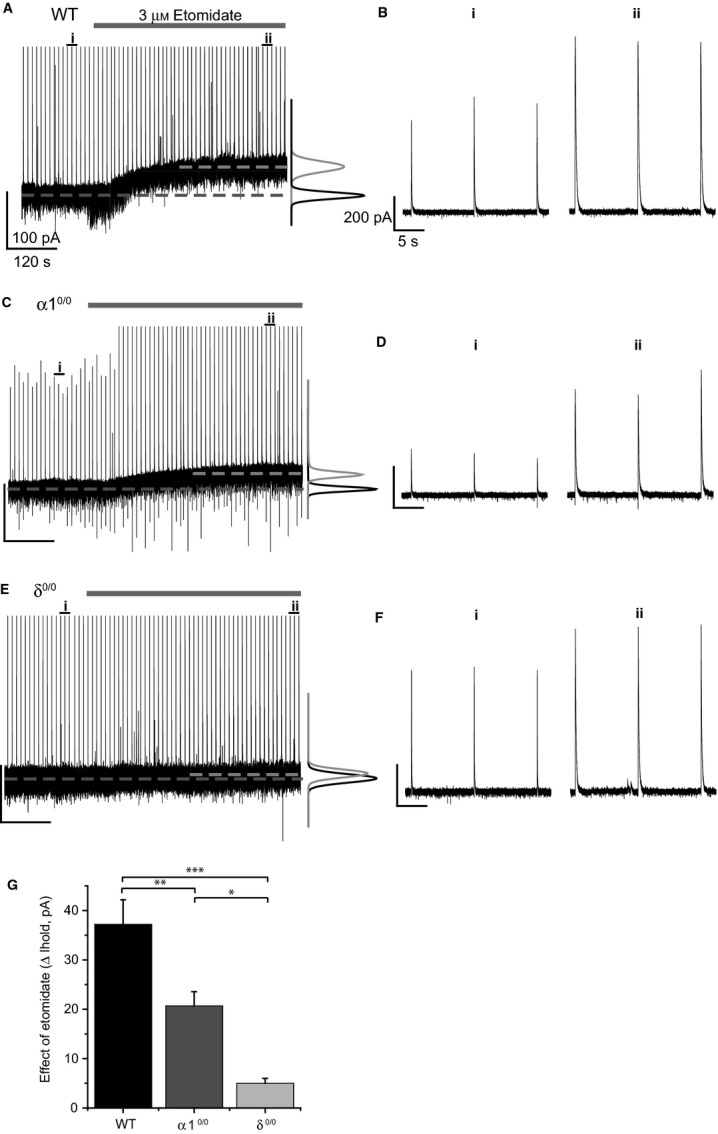
Deletion of the α1 or δ subunit reduces the effect of etomidate on VB tonic inhibition. (A, C, E) Representative whole-cell recordings (left) and corresponding all points histograms (right), illustrating the effect of etomidate on the holding current of WT (A), α1^0/0^ (C) and δ^0/0^ (E) VB neurons. Evoked IPSCs were simultaneously monitored throughout the recordings, but have been truncated to emphasize changes in holding current (*I*_hold_). Non-truncated eIPSCs denoted by i (control) and ii (etomidate) are shown in B, D and F for WT, α1^0/0^ and δ^0/0^, respectively, to confirm the effect of etomidate on eIPSCs. (G) Bar graph comparing the outward current induced by the bath application of etomidate (expressed as a change in *I*_hold_) for WT (black bar, *n* = 8), α1^0/0^ (dark grey bar, *n* = 9) and δ^0/0^ (light grey bar, *n* = 7) VB neurons. *P <* *0.05, **P <* *0.01, ***P <* *0.001, one-way anova, Tukey's *post hoc* test.

While quantifying the effects of etomidate on eIPSCs, we simultaneously monitored the VB holding current in each mouse strain to further understand the contribution of α1βγ2 and α4βδ GABA_A_Rs to the effects of the anaesthetic on VB tonic inhibition. Bath application of 3 μm etomidate induced a clear outward shift in VB holding current (37.2 ± 5.0 pA, *n* = 8; Fig.5A and G), with a time course similar to that observed for potentiation of eIPSCs (Fig.5B, panels i and ii). In previous work, we found that deletion of α1-containing receptors did not significantly influence baseline tonic current amplitudes, consistent with the hypothesis that VB tonic inhibition is mediated predominantly by α4β2δ receptors (Chandra *et al*., 2006; Herd *et al*., 2009). Thus, it was surprising that while etomidate application induced a clear outward current in α1^0/0^ VB recordings (20.7 ± 2.9 pA, *n* = 9) concurrently with potentiation of residual eIPSCs (Fig.5C and D), the magnitude of the outward current shift was significantly reduced relative to WT (Fig.5G, drug × genotype interaction, *F*_2,21_ = 20.11, *P *<* *0.001, mixed anova). In recordings from VB neurons of δ^0/0^ mice, bath application of 3 μm etomidate induced only a minimal outward current (5.0 ± 1.0 pA, *n* = 7; *P *<* *0.001 vs. WT; P = 0.012 vs. α1^0/0^, mixed anova, Tukey's post-hoc test; Fig.5E and G), while robustly potentiating eIPSCs (Fig.5F).

### Deletion of α1 or δ subunits attenuates the effect of etomidate on VB neuron excitability

**Figure 6:**
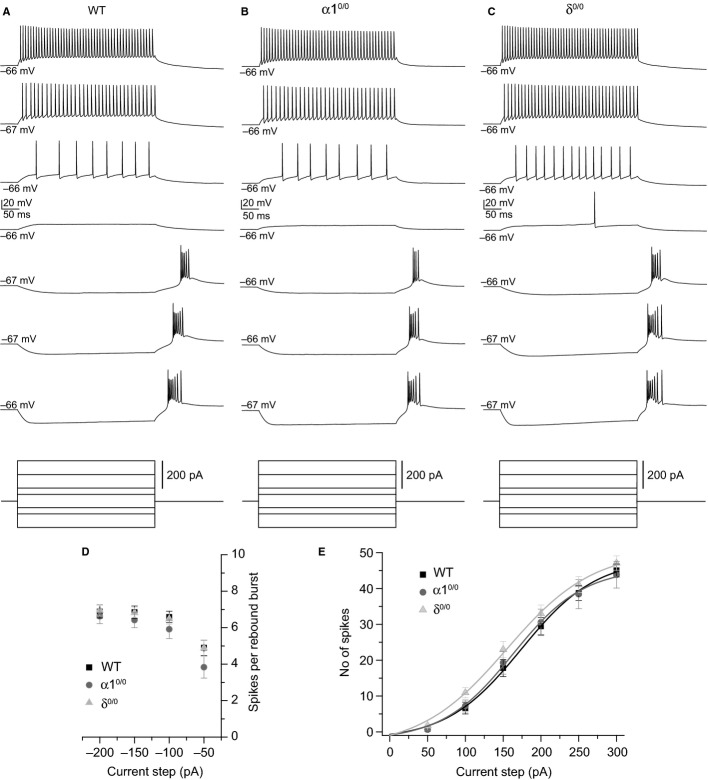
The excitability of VB neurons is not significantly altered in α1^0/0^ or δ^0/0^ mice. (A–C) Whole-cell current-clamp recordings from WT (A), α1^0/0^ (B) and δ^0/0^ (C) VB neurons, illustrating voltage responses to a family of hyperpolarizing and depolarizing current steps (−200 to +300 pA, 50 pA increments). For clarity, the responses to only a selection of tested current steps are shown, as indicated in the stimulation protocol (bottom). (D) Graph summarizing the number of spikes occurring within rebound bursts upon offset from hyperpolarizing current steps in recordings from WT (black squares, *n* = 23), α1^0/0^ (dark grey circles, *n* = 12) and δ^0/0^ (light grey triangles, *n* = 22) VB neurons. (E) Graph comparing the number of spikes generated in response to depolarizing current steps of increasing amplitude in VB neurons derived from WT, α1^0/0^ and δ^0/0^ mice. Symbols and *n* numbers are as indicated for D. The input–output relationship for each strain is fitted with a Boltzmann sigmoid curve.

Given that etomidate modulation of VB tonic inhibition is significantly reduced in α1^0/0^ and δ^0/0^ neurons, such genetic manipulations are thus predicted to influence the effect of the anaesthetic on cellular excitability. To investigate this hypothesis, we examined the effect of etomidate on VB input–output (I–O) relationships generated in response to a family of current steps (−200 to +300 pA, 400 ms duration, 50-pA increments every 15 s). Current steps were delivered while neurons were held at a membrane potential of ∼−66 mV; at such potentials, *I*_T_ is largely inactivated (Coulter *et al*., 1989). First, we compared I-O relationships across WT, α1^0/0^ and δ^0/0^ under control conditions to determine if basic electrical excitability was affected by the mutations (Fig.6). Across all three strains, in accordance with classical TC neuron physiology, hyperpolarizing currents steps of sufficient amplitude generated a sagging, hyperpolarization-activated cation current (*I*_H_)-dependent membrane potential response, followed by a rebound low-threshold Ca^2+^ potential, crowned by a high-frequency spike burst (spikes per burst, WT = 2–10, *n* = 23; α1^0/0^ = 1–9, *n* = 12; δ^0/0^ 2–10, *n* = 22). Although the amplitude of the hyperpolarizing current step significantly influenced the mean number of spikes per rebound burst (*F*_3,162 = _104.80, *P *<* *0.001, mixed anova), there was no significant effect of genotype on this relationship (*F*_6,162_ = 1.00, *P *= 0.43, mixed anova, Fig.6D). Supra-threshold depolarizing current steps of increasing amplitude generated tonic spikes of increasing frequency in VB neurons derived from each mouse strain (Fig.6A–C and E). Although visual inspection of the resulting I-O curves (Fig.6E) suggests a modest left-ward trend of the relationship towards increased excitability for δ^0/0^ relative to WT recordings, this did not reach statistical significance (*F*_5,215_ = 1.46, *P = *0.20, mixed anova). Indeed, estimation of the current amplitude generating half maximal spike output (EA_50_) from Boltzmann sigmoidal curve fits (see Methods) indicated a small, but non-significant effect of δ subunit deletion on the I-O EA_50_ (WT = 173 ± 7 pA, *n* = 23; α1^0/0^ = 168 ± 10 pA, *n* = 12; δ^0/0^ = 158 ± 8 pA, *n* = 22, *F*_2,54_ = 1.00, *P *= 0.37, one way anova). Similarly, neither the resting membrane potential nor IR of VB neurons was significantly influenced by deletion of the δ subunit, although again a trend towards an increased IR was observed relative to WT (IR, WT = 288 ± 13 MΩ, *n* = 38; δ^0/0^ = 328 ± 20 MΩ, *n* = 27, *P* = 0.1, unpaired *t*-test, Table3).

**Figure 7:**
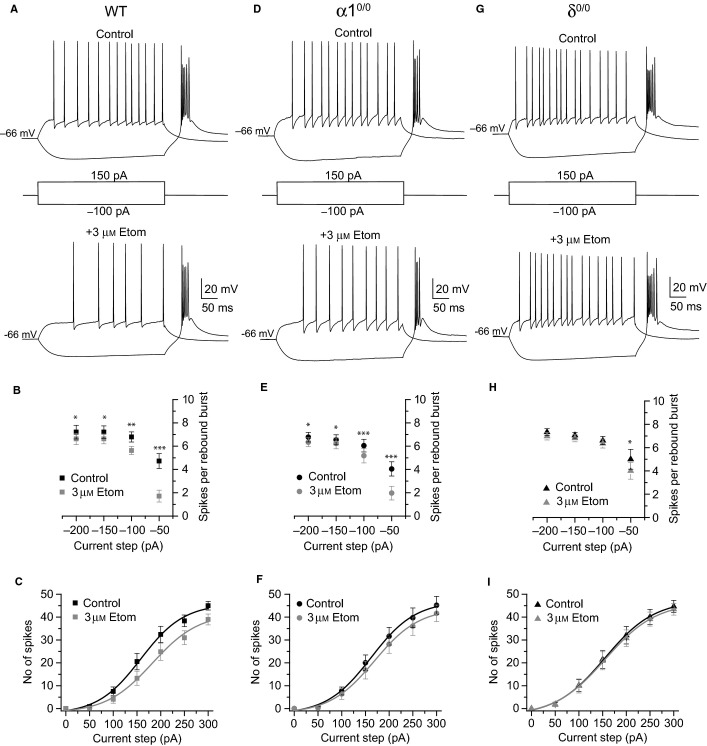
The effect of etomidate on VB neuron excitability is attenuated by deletion of the α1 subunit and abolished by deletion of the δ subunit. (A) Representative whole-cell current-clamp recording from a WT VB neuron, illustrating superimposed voltage responses to the indicated current steps (middle) under control conditions (top) and in the presence of 3 μm etomidate (bottom). (B) Graph comparing the intra-burst spike number observed on rebound from a range of hyperpolarizing current steps (−200 to −50 pA), before (black symbols) and after (grey symbols) etomidate (*n* = 12). (C)Graph comparing the number of spikes generated in response to depolarizing current steps of increasing amplitude (50–300 pA) under control conditions (black symbols), and in the presence of etomidate (grey symbols). (D–F) Effect of etomidate on the excitability of α1^0/0^ VB neurons (*n* = 11). Figure details are as described for A–C. (G–I) Effect of etomidate on the excitability of δ^0/0^ VB neurons (*n* = 9). Figure details are as described for A–C. The input–output relationships determined for each condition are fitted with a Boltzmann curve. *P <* *0.05, **P <* *0.01, ***P <* *0.001, paired *t*-test. Etom, etomidate.

In accordance with the large potentiation of tonic inhibition, bath application of 3 μm etomidate hyperpolarized the resting *V*_m_, reduced membrane IR and exerted a clear reduction in the excitability of WT VB neurons (Table4, Fig.7A–C). Thus, etomidate significantly reduced the number of spikes per rebound burst upon release from hyperpolarizing stimulations, although the effect was particularly evident for smaller amplitude current steps (Fig.7A and B). Similarly, etomidate reduced the tonic spike output, resulting in a clear rightward shift of the I-O relationship of VB neurons (Fig.7A and C). Indeed, the EA_50_ determined from Boltzmann fits to I-O curves was significantly increased in the presence of the anaesthetic (EA_50_: control = 170 ± 12 pA; + etomidate = 195 ± 12 pA, *n* = 12, *P *<* *0.001). By contrast, although etomidate also hyperpolarized the resting *V*_m_ and decreased the membrane IR of α1^0/0^ VB neurons (Table4), the consequent reduction of their excitability was less than for WT neurons (Fig.7D–F). These findings are consistent with the reduced enhancement of tonic inhibition by the anaesthetic following α1 subunit deletion. This was particularly evident upon inspection of the I-O relationship generated from depolarizing stimulations, where the rightward shift in the curve induced by etomidate (EA_50_: control = 166 ± 11 pA, + etomidate = 178 ± 13 pA, *n* = 11, *P *= 0.03) was significantly reduced relative to WT recordings (Table5). Deletion of the δ subunit virtually abolished the effect of etomidate on VB burst and tonic firing (Fig.7G–I), consistent with the limited etomidate-induced outward current observed in δ^0/0^ voltage-clamp experiments. In addition, for δ^0/0^ neurons, etomidate had minimal effect on the resting *V*_m_, or on the membrane input resistance (Table4). Thus, with the exception a small effect at the smallest current step, which was significantly reduced relative to that observed in WT recordings (*P = *0.019 unpaired *t*-test), etomidate did not significantly influence rebound intra-burst spike number in response to hyperpolarizing current steps (Fig.7H). Similarly, for δ^0/0^ neurons, the I-O curve generated in response to positive current steps remained relatively unaffected by etomidate (EC_50_: control = 163 ± 14 pA, + 3 μm etomidate = 163 ± 15 pA, *n* = 9, *P *= 0.95, Fig.7I; Table5).

**Table 4:** Comparison of the effect of etomidate on the resting membrane potential (*V*_m_) and input resistance of WT, α1^0/0^ and δ^0/0^ VB neurons

	WT (*n *=* *19)		α1^0/0^ (*n *=* *12)		δ^0/0^ (*n *=* *11)	
	Control	3 μm etomidate	Control	3 μm etomidate	Control	3 μm etomidate
*V*_m_ (mV)	–71.7 ± 0.8	–73.2 ± 0.9***	–71.7 ± 0.8	–73.2 ± 0.9***	–74.6 ± 0.6	–75.5 ± 0.7*
Input resistance	258 ± 14	193 ± 11***	285 ± 26	247 ± 23**	287 ± 31	275 ± 27

* *P*<0.05

** *P*<0.01

*** *P*<0.001

paired *t*-test.

**Table 5:** Comparison of the effect of etomidate on the input–output relationship of WT, α1^0/0^ and δ^0/0^ VB neurons

	Etomidate-induced change in EA_50_ (pA)
WT (*n *=* *12)	25 ± 4
α1^0/0^ (*n *=* *11)	12 ± 5*
δ^0/0^ (*n *=* *19)	0 ± 6**

* *P *<0.05

** *P *<0.05

vs. WT, one-way anovaTukey's post-hoc test.

## Discussion

In this study, we investigated the relative contribution of synaptic and extrasynaptic GABA_A_Rs to the etomidate-induced inhibition of neuronal activity in a network putatively involved in general anaesthetic mechanisms. Specifically, deletion of the δ subunit reduced the effect of etomidate on VB tonic inhibition. Consequently, the ability of etomidate to inhibit burst and tonic spiking was virtually abolished in δ^0/0^ mice, suggesting that etomidate facilitation of tonic inhibition exerts a dominant effect on TC cell output. Unexpectedly, deletion of the α1 subunit also reduced the impact of etomidate on VB tonic inhibition and the relay neuron I-O relationship, thus implicating the α1-GABA_A_R population during anaesthetic actions in thalamus. Importantly, etomidate also greatly prolonged the duration of nRT burst-mediated phasic inhibition, by potentiating both synaptic and extrasynaptic GABA_A_Rs. Thus, further to conventional phasic and tonic inhibition, burst-mediated spill-over may represent a novel mechanism whereby anaesthetics can modulate circuit function.

While anaesthetics probably exert their behavioural effects by altering neuronal activity across multiple brain regions (Franks, 2008), human neuro-imaging studies have identified a consistent thalamic deactivation during anaesthetic-induced sedation and/or loss of consciousness (Fiset *et al*., 1999; Alkire *et al*., 2000; White & Alkire, 2003; Hofbauer *et al*., 2004; Gili *et al*., 2013). Such studies are consistent with the canonical view of the thalamus as a sensory ‘gate’ that governs the transmission of multi-modal information to the cortex (Steriade, 2003). *In vivo*, anaesthetics may modulate thalamic activity directly by influencing the intrinsic excitability of resident thalamic neurons or indirectly by altering the activity of extrinsic excitatory inputs (e.g. descending cortico-thalamic, or ascending reticular activating system inputs). Indeed, single-unit recordings from cats showed a reduction of the spontaneous tonic firing rate of TC neurons by etomidate, alongside similar decreases in the neocortex and reticular formation (Andrada *et al*., 2012). Computer simulations utilizing thalamocortical network models have also provided important insights, revealing that elevated inhibition of cortical and thalamic neurons imposes a slowed, hyper-synchronous rhythmicity upon the thalamocortical loop that is reminiscent of electroencephalography dynamics during anaesthetized states (Talavera *et al*., 2009; Ching *et al*., 2010; Purdon *et al*., 2013). Collectively, such studies favour a co-ordinated action of anaesthetics across key interconnected networks, rather than a specific localized effect. Nevertheless, our data support the hypothesis that etomidate mediates at least a component of its actions by a direct reduction of TC neuron output, resulting from enhanced synaptic and extrasynaptic GABA_A_R function. In agreement, propofol and the volatile agent isoflurane also inhibit relay neuron tonic spiking in a manner that is at least partially GABA_A_R-dependent (Ying & Goldstein, 2005a,b2005b; Jia *et al*., 2008).

That some general anaesthetics exert their spectrum of behavioural effects via a selective interaction with the GABA_A_R is well established (Hales & Lambert, 1991; Franks & Lieb, 1994). In support, mutant mice harbouring point-mutated GABA_A_Rs engineered to be etomidate-insensitive (β2N265S; Belelli *et al*., 1997; Reynolds *et al*., 2003) or propofol- *and* etomidate-insensitive (β3N265M; Pistis *et al*., 1999; Jurd *et al*., 2003) display dramatically altered behavioural responses to these anaesthetics, revealing that specific anaesthetic behaviours can be assigned to individual GABA_A_R populations. Specifically, the sedative and immobilizing effects of both etomidate and propofol are mediated by β2- and β3-containing receptors, respectively, whereas both receptor populations contribute to the hypnotic component (Jurd *et al*., 2003; Reynolds *et al*., 2003). However, these studies did not address which β2- or β3-expressing neuronal populations may be involved in the respective behaviours. Thus, it is noteworthy that the effects of etomidate on relay cell phasic and tonic currents are greatly reduced in β2N265S slices (Belelli *et al*., 2005). In light of the present evidence for a reduced etomidate-induced inhibition of relay neuron spiking in α1^0/0^ and δ^0/0^ mice, such studies implicate synaptic (α1β2γ2) and extrasynaptic (α4β2δ) GABA_A_Rs as important molecular targets for the thalamic actions of this anaesthetic.

The effect of etomidate on VB tonic inhibition was virtually abolished in δ^0/0^ mice, leading to a reduced inhibitory effect on burst and tonic spiking. Such observations are consistent with the tonic conductance generating an inhibitory charge which greatly outweighs that produced by brief, spontaneous phasic inhibition. In agreement, computational studies utilizing a thalamocortical network model suggest that enhanced tonic inhibition is critical for the etomidate-induced depression of thalamic firing (Talavera *et al*., 2009). Thus, the significant effect of the α1 subunit deletion (albeit lesser than for the δ^0/0^) was unexpected. One potential interpretation suggests that a subpopulation of δ-GABA_A_Rs may contain the α1 subunit instead of α4. Indeed, α1 can form a functional partnership with the δ subunit in some hippocampal interneurons (Glykys *et al*., 2007). However, while α1 subunit deletion abolishes spontaneous phasic inhibition in VB (Peden *et al*., 2008), we did not detect a significant reduction of baseline tonic inhibition, or the current induced by the δ-selective agonist THIP (4,5,6,7-tetrahydroisoxazolo[5,4-c]pyridin-3-ol), which does not discriminate between α1βδ and α4βδ receptors (B. Ebert, personal communication; Herd *et al*., 2009), suggesting that α1βδ receptors are not abundantly expressed in VB neurons. Alternatively, the loss of on-going spontaneous phasic inhibition, which is robustly prolonged by etomidate in WT neurons, may partially account for the reduced effect of the anaesthetic on spike discharge for α1^0/0^ relay neurons. Additionally, a substantial pool of α1βγ2 receptors may exist extrasynaptically (Kasugai *et al*., 2010). While this receptor population does not appear to contribute to baseline tonic inhibition, etomidate, which increases GABA_A_R channel open probability in response to low GABA concentrations (Yang & Uchida, 1996), may enhance the activation of extrasynaptic α1β2γ2 receptors by ambient GABA. Finally, we cannot exclude that direct activation of α1β2γ2 receptors by etomidate (Hill-Venning *et al*., 1997) may influence VB neuron excitability.

Using paired nRT–VB recordings, we recently reported that peri/extrasynaptic α4βδ receptors are recruited during nRT burst firing, leading to a substantial prolongation of burst-mediated phasic inhibition (Herd *et al*., 2013). In support of this, despite the absence of synaptic GABA_A_Rs, we observed a residual eIPSC in α1^0/0^ VB neurons, which was greatly potentiated by the δ-selective positive allosteric modulator DS2 (Herd *et al*., 2013). Here, we observed a similar potentiation of the residual eIPSC upon application of etomidate to α1^0/0^ slices. Consequently, the remarkable ability of the residual eIPSPs to suppress tonic spiking of α1^0/0^ neurons was greatly prolonged by etomidate. In α4^0/0^ and δ^0/0^ mice, eIPSCs are shorter due to the abolished spill-over component, and are insensitive to DS2, consistent with the loss of α4βδ receptors (Herd *et al*., 2013). In contrast to DS2, in δ^0/0^ recordings, etomidate significantly prolonged eIPSCs and therefore the eIPSP-induced suppression of tonic spiking. Curiously, for the latter paradigm, the duration of spike suppression induced by the eIPSP in the presence of etomidate was not significantly different from WT in either α1^0/0^ or δ^0/0^ neurons (Fig.3D), despite a clear effect of both gene deletions under control conditions (Fig.2D). In current-clamp recordings the duration of IPSPs is determined partially by the kinetic properties of GABA_A_Rs, but also by the ensuing activation of voltage-dependent conductances (e.g. *I*_H_ and *I*_T_), which may act to truncate the IPSP time course (Thomson *et al*., 2000). Thus, we speculate that the duration of the IPSP may be maximized in the presence of the anaesthetic and that this ‘ceiling effect’ may be achieved via the potentiation of either synaptic or extrasynaptic GABA_A_Rs in the absence of the complementary receptor population. Nevertheless, the results suggest that etomidate facilitates burst-mediated inhibition via a combined action at synaptic and peri/extrasynaptic GABA_A_Rs, leading to an amplified inhibitory effect on VB tonic firing.

As noted above, the strength of synaptic inhibition arising from nRT neurons interacts with intrinsic voltage-dependent conductances to control the timing of post-inhibitory rebound bursts, and thus the period and synchrony of thalamus-dependent oscillations, which prevail in the unconscious brain (Huguenard & McCormick, 2007). In addition to an enhanced tonic inhibition, which induces a membrane hyperpolarization that is likely to favour burst firing (Cope *et al*., 2005), etomidate also delayed the timing of post-inhibitory rebound burst firing by prolonging the IPSP. Given the importance of burst firing during thalamocortical rhythmogenesis, the etomidate-induced increase of IPSP duration may therefore contribute to the slowed oscillatory activity of the anaesthetized brain.

Our *in vitro* data, together with a computational study (Talavera *et al*., 2009), suggest that the etomidate-induced inhibition of TC output is dependent on synaptic and extrasynaptic GABA_A_Rs. Thus, it is surprising that deletion of α1, α4 or δ subunits exerts little or no influence on the sedative or hypnotic actions of etomidate (Mihalek *et al*., 1999; Kralic *et al*., 2003; Iyer *et al*., 2013). However, the interpretation of behavioural data obtained from mice harbouring global GABA_A_R subunit deletions is complicated by the non-uniform up-regulation of alternative GABA_A_R isoforms following subunit loss (Sur *et al*., 2001; Kralic *et al*., 2002a,b2002b; Peng *et al*., 2002). This caveat is exemplified by studies investigating the subunit-dependence of benzodiazepine-induced behaviours; mice expressing a benzodiazepine-insensitive α1 subunit (H101R) are resistant to the sedative effects of diazepam (Rudolph *et al*., 1999; McKernan *et al*., 2000), yet α1^0/0^ mice exhibit enhanced diazepam-induced sedation (Kralic *et al*., 2002b). Thus, caution is warranted when comparing results from a slice preparation demonstrating minimal functional GABA_A_R compensation to behavioural studies underpinned by complex interactions between multiple brain networks subject to varying degrees of compensation. We note that an as yet unidentified neuronal adaptation (e.g. as described by Brickley *et al*., 2001) is likely to compensate for the loss of tonic or phasic inhibition in relay neurons, as demonstrated by the lack of any significant alteration in membrane IR or the I-O relationship in the mutant mice. Crucially, however, such adaptations do not appear to include upregulation of the remaining palette of GABA_A_R subunits (Kralic *et al*., 2006; Peden *et al*., 2008; Herd *et al*., 2009).

In summary, we have shown that etomidate reduces the electrical excitability of thalamocortical relay neurons via a specific interaction with synaptic and extrasynaptic GABA_A_Rs. Given the established role of the thalamus in arbitrating conscious state transitions, our results support the putative role of these distinct thalamic GABA_A_R populations in general anaesthetic actions. Finally, we suggest that burst-mediated spill-over, a kinetically intermediate form of inhibition that bridges the kinetic extremes of fast phasic and slow tonic inhibition, represents a novel means by which GABA-modulatory anaesthetics may influence the relay of sensory information from thalamus to cortex.

## Abbreviations

anova: analysis of variance
DMSO: dimethyl sulphoxide
DS2: δ-selective compound 2
EA_50_: excitatory current required for half maximal spike frequency
ECS: extracellular solution
eGABA_A_R: extrasynaptic γ-aminobutyric acid, type A receptor
eIPSC: evoked IPSC
eIPSP: evoked IPSP
*E*_rev_: reversal potential
GABA: γ-aminobutyric acid
GABA_A_R: γ-aminobutyric acid, type A receptor
GABA_B_: γ-aminobutyric acid, type B receptor
*I*_H_: current mediated by hypepolarization-activated, cyclic nucleotide-gated cation channels
I-O: input–output relationship
IPSC: inhibitory postsynaptic current
IPSP: inhibitory postsynaptic potential
IR: membrane input resistance
ISI: inter-spike interval
*I*_T_: current mediated by low threshold T-type Ca^2+^ channels
mIPSC: miniature IPSC
nRT: nucleus reticularis thalami
TC: thalamocortical
VB: ventrobasal complex
*V*_m_: membrane potential
WT: wild-type
α1^0/0^: α1 subunit knock-out mouse
α1-GABA_A_R: α1 subunit-containing GABA_A_ receptor
β3N265M: asparagine to methionine mutation at position 265 of the GABA_A_ receptor β3 subunit
β2N265S: asparagine to serine mutation at position 265 of the GABA_A_ receptor β2 subunit
δ^0/0^: δ subunit knock-out mouse
δ-GABA_A_R: δ subunit-containing GABA_A_ receptor
τ_w_: weighted decay time constant
